# Prevention of Delirium Via Melatonin and Orexin Neurotransmission

**DOI:** 10.14789/jmj.JMJ21-0035-R

**Published:** 2022-02-04

**Authors:** KOTARO HATTA

**Affiliations:** 1 Department of Psychiatry, Juntendo University Nerima Hospital, Tokyo, Japan; 1 Department of Psychiatry, Juntendo University Nerima Hospital, Tokyo, Japan

**Keywords:** delirium, melatonin, orexin, prevention, sleep-wake cycle disturbance

## Abstract

The fundamental conception of delirium is altered arousal. In addition, sleep-wake cycle isturbances including insomnia, excessive daytime napping, and disintegration of the xpected circadian patterns have been described as a characteristic component of delirium or decades, and demonstrated to be a core symptom domain of delirium. Although on-pharmacological interventions are successful to some extent, they have limitations due o various biological etiologies for delirium. Among pharmacological interventions, ntipsychotics seem to be effective, but they are not suitable for preventive use because f relatively frequent side-effects such as extrapyramidal symptoms. Recently, new type of rugs for insomnia have been focused with respect to delirium prevention. Recent eta-analyses show effectiveness of melatonin receptor agonists and orexin receptor ntagonists for delirium prevention, and real-world data support them.

## 1. Introduction

The fundamental conception of delirium is altered arousal^1)^. In addition, sleep-wake cycle disturbances including insomnia, excessive daytime napping, and disintegration of the expected circadian patterns have been described as a characteristic component of delirium for decades, and demonstrated to be a core symptom domain of delirium^2)^. Although non-pharmacological interventions are successful to some extent^3)^, they have limitations due to various biological etiologies for delirium. Among pharmacological interventions, antipsychotics seem to be effective, but they are not suitable for preventive use because of relatively frequent side-effects such as extrapyramidal symptoms. Recently, new type of drugs for insomnia have been focused with respect to delirium prevention.

## 2. Melatonin receptor agonists for delirium prevention

Melatonin, a pineal gland hormone, regulates the sleep-wake cycle, and there is some emerging literature suggesting that melatonin prophylaxis may reduce delirium incidence or a long-lasting episode of delirium^4-6)^. Sultan reported that after medications were given orally 90 min before operative time and at sleep time at night of operation, the melatonin group showed a statistically significant decrease in the percentage of postoperative delirium to 9.43% (5/53 patients), compared with the control group (32.65% [16/49], relative risk 0.29, *P* = .0062)^4)^. Al-Aama et al. reported that melatonin (0.5 mg every night for 14 days or until discharge) was associated with a lower risk of delirium (12.0% vs. 31.0% [placebo], *P* = .014), with an odds ratio (OR), adjusted for dementia and co-morbidities of 0.19^5)^. de Jonghe et al. reported that despite no effect of melatonin (3 mg in the evening for 5 consecutive days) on the incidence of delirium (29.6% [55/186 patients scheduled for acute hip surgery] for the melatonin group vs. 25.5% [49/192] for the placebo group), a smaller proportion of patients in the melatonin group than in the placebo group experienced a long-lasting episode of delirium (> 2 days) (25.5% v. 46.9%; *P* = .02)^6)^.

Along the similar conception, we examined whether ramelteon, a melatonin agonist with 6- and 3-fold higher affinities for melatonin 1 (MT_1_) and melatonin 2 (MT_2_) receptors, respectively, compared to those of melatonin, is effective for the prevention of delirium in a multi-center, rater-blinded, randomized placebo-controlled clinical trial^7)^. Eligible patients were 65-89 years old, newly admitted to intensive care units and regular acute wards in four university hospitals and one general hospital due to emergency, and able to take medicine orally. Patients were excluded from the study if they had an expected stay or life expectancy less than 48 h. Sixty-seven patients were randomly assigned using the sealed envelope method to receive ramelteon (8 mg/day; n=33) and placebo (n=34) every night for 7 days, and the main outcome measure was incidence of delirium as determined by the DSM-IV-TR. Ramelteon was associated with lower risk of delirium (3% vs. 32%, *P* =.003), with a relative risk of 0.09 (95% confidence interval [CI], 0.01-0.69). Even after controlling for risk factors such as age, diagnosis of dementia, and admission diagnosis of infection, ramelteon was still associated with a lower incidence of delirium (*P* =.01; odds ratio, 0.07; 95%CI, 0.008-0.54). Kaplan-Meier estimates of time to development of delirium were 6.94 days (95%CI, 6.82-7.06 days) for ramelteon and 5.74 days (5.05-6.42 days) for placebo. Comparison by log-rank test showed that the frequency of developing delirium was significantly lower in patients taking ramelteon than in those taking placebo (*χ*^2^=9.83, *P* =.002). Furthermore, ramelteon was associated with lower risk of delirium among patients with the Clinical Dementia Rating (CDR) ≥0.5 (the ramelteon group, 6% vs. the placebo group, 62%, *P* =.003), with a relative risk of 0.15 (95% CI, 0.02-0.96)^8)^. Thus, these findings suggest that ramelteon administered nightly to elderly patients admitted for acute care provides protection against delirium, and support a possible pathogenic role of melatonin neurotransmission in delirium. One limitation in clinical practice, especially in an intensive care situation, is the lack of intravenous formulations of melatonin and its agonists.

Another possibility of mechanism for preventive effects of melatonin and ramelteon on delirium other than improvement in sleep-wake cycle is anti-microbial properties. Fink et al. reported that survival rate in rats experimentally having developed sepsis was significantly improved after administration of ramelteon or melatonin 1.0 mg/kg, compared with vehicle-treated animals, and that coadministration of melatonin receptor-antagonist luzindole abolished this effect completely^9)^. As infection is a clinical factor that might precipitate delirium^10)^, anti-septic effects of melatonin and ramelteon might be associated with the preventive effects on delirium. This finding can be partially explained through change in the balance between two pathways of tryptophan metabolism under inflammation. Tryptophan is metabolized through two major pathways, the kynurenine pathway and the methoxyindole pathway. The methoxyindole pathway generates serotonin, which is a further substrate for melatonin biosynthesis. The kynurenine pathway can be activated as a result of inflammatory stimuli^11)^. This change in the balance between two pathways of tryptophan metabolism can decrease in melatonin biosynthesis. Therefore, administration of melatonin or ramelteon under systemic inflammation, which may cause delirium, can compensate for lack of melatonin, resulting in the prevention of delirium.

Recent meta-analysis shows effectiveness of melatonin receptor agonists for delirium prevention^12)^.

## 3. Orexin receptor antagonists for delirium prevention

Orexin is an alerting neuropeptide produced by neurons located predominantly in lateral hypothalamic area, perifornical area and posterior hypothalamus^13)^. The orexin-A levels in cerebrospinal fluid (CSF) during active wake state are highest during the dark phase in nocturnal rodents and highest during the light phase in diurnal species^14)^. As a primary arousal signal in wake control, orexin signaling is necessary for normal circadian regulation of consolidated wakefulness. Accordingly, as compared to melatonin, orexin is predominantly secreted during daytime in humans^13)^. In contrast to melatonin, it has been reported that orexin secretion decreases by 10% only in older adults as compared to younger adults^15)^.

Interestingly, as compared to controls, patients with moderate to severe Alzheimer disease were shown to have higher mean orexin levels in CSF. Additionally, these patients had significantly impaired nocturnal sleep compared to controls and patients with mild Alzheimer’s disease^16)^. Furthermore, significantly higher levels of orexin in the brain of rats with acute pancreatitis were reportedly found when compared to healthy controls^17)^. As dementia and inflammation are risk factors for delirium, it is possible that patients with delirium have increased orexin levels, thus accounting for the resultant sleep-wake disturbance^18)^.

Suvorexant, a potent and highly selective for orexin-1 receptor and orexin-2 receptor antagonist receptor antagonist, is an FDA approved treatment for primary insomnia in U.S. Suvorexant has been associated with improvements in subjective measures of total sleep time (sTST), time to sleep onset (sTSO), and wake after sleep onset (sWASO)^19)^, without altering NREM and REM sleep architecture as assessed by electroencephalographic monitoring^20)^. The scientific rationale for using suvorexant for delirium prevention was that such character of suvorexant promoting natural sleep could improve sleep-wake cycle disturbance in delirium. High selectivity for orexin-1 and orexin-2 receptor antagonism^21)^ and little affinity for acetylcholine receptors (Ki>10µM)^22)^ may be advantage for delirium prevention. Reportedly, another dual orexin receptor antagonist did not lower hippocampal acetylcholine^23)^. Thus, we hypothesized that suvorexant would have effects of preventing delirium.

To assess the efficacy of suvorexant in prevention of delirium, our group conducted a multi-center, rater-blinded, placebo-controlled RCT in ICUs and acute-phase wards. Eligible patients were aged 65-89 years, who were newly admitted due to medical and surgical emergency, able to take medicine orally, and expected to stay less than 48 hours. Study participants were randomly assigned to suvorexant (15 mg/day) (n=36) or placebo (n=36) nightly for 3 days. Main outcome measure was incidence of delirium according to the DSM-5. Patients taking suvorexant developed delirium less frequently than those taking placebo (suvorexant, 0% (n/N = 0/36) vs. placebo, 17% (6/36), P = 0.025)^24)^. With respect to changes in sleep-wake cycle disturbance score (item #1) of DRS-R-98, analysis of variance (ANOVA) revealed a tendency for main effect of treatment, suggesting the potential of suvorexant to improve sleep-wake cycle disturbance that is a core feature of delirium.

Another RCT examining the effects suvorexant on delirium prevention in ICU setting showed that, as compared to placebo, suvorexant led to significant reduction in incidence of both clinical delirium (14.7% vs. 33.3%, P = 0.069) and sub-syndromal delirium symptoms (17.6% vs. 47.2%, P = 0.011)^25)^. Furthermore, Kaplan-Meier estimates revealed that time to delirium onset was significantly longer in the suvorexant group as compared to the placebo group. These findings, taken together, suggest that suvorexant, a potent and selective orexin antagonist, has beneficial effects on delirium prevention, and highlight the importance of correcting sleep-wake cycle disturbance in prevention of delirium.

Recent meta-analysis shows effectiveness of orexin receptor antagonists for delirium prevention^26)^.

## 4. Real-world effectiveness of melatonin receptor agonists and orexin receptor antagonists for delirium prevention

After success of RCTs on delirium prevention by ramelteon and suvorexant, we choose these drugs for patients who have risk factors for delirium in clinical practice. So, we examined whether ramelteon and/or suvorexant would affect delirium prevention among both patients at risk for but without delirium (patients-at-risk), and those with delirium (patients-with-delirium) on the night before a consultation. This multicenter, prospective, observational study was conducted by trained psychiatrists as consultation-liaison psychiatric services between October 2017 and October 2018. Patients who were age 65 years or older and hospitalized because of acute diseases or elective surgery, had risk factors for delirium, and had insomnia or delirium on the night before the consultation were prescribed ramelteon and/or suvorexant. The decision to take medication was left to the discretion of each patient. The primary outcome was incidence of delirium based on the Diagnostic and Statistical Manual of Mental Disorders, Fifth Edition, during the first 7 days. Among 526 patients-at-risk, those taking ramelteon and/or suvorexant developed delirium significantly less frequently than those who did not, after controlling for the effects of risk factors on the estimate of an independent association between the effects of ramelteon and/or suvorexant and the outcome of developing delirium (15.7% vs. 24.0%; odds ratio [OR]: 0.48, 95% confidence interval [CI]: 0.29–0.80, P = 0.005)^27)^. Similar results were found among 422 patients-with-delirium (39.9% vs. 66.3%; OR: 0.36, 95%CI: 0.22–0.59; P < 0.0001). Thus, ramelteon and suvorexant appears to be effective for delirium prevention in real-world practice.

## 5. Conclusion

According to the evidence, we choose ramelteon first, and orexin receptor antagonists second for insomnia in elderly patients to prevent developing delirium. Thus, a potent melatonin agonist ramelteon and orexin receptor antagonists play important roles not only in insomnia but also in the prevention of delirium.

## Funding

This work was supported by the JSPS KAKENHI Grant Number 20K07927. The sponsor of the study had no role in the study design, data collection, data analysis, data interpretation, writing of the report, or decision to submit the paper for publication.

## Author contributions

Kotaro Hatta designed the study, obtained funding, analyzed the data, interpreted the data, drafted the report, and contributed to and have approved the final manuscript.

## Conflicts of interest statement

Dr Hatta has received lecture honoraria for Dainippon-Sumitomo, Eisai, Janssen, Meiji Seika, MSD, and Otsuka.
